# Towards sustainable 6G: A collaborative call to action for addressing environmental challenges in (and thanks to) future mobile networks

**DOI:** 10.12688/openreseurope.18767.1

**Published:** 2024-12-09

**Authors:** Regis Decorme, Sébastien Faye, Monique Calisti, Simon Pryor, Indrakshi Dey, Maria Pia Fanti, Francesco Malandrino, Chiara Lombardo

**Affiliations:** 1 R2M Solution, Roquefort-les-Pins, 06330, France; 2 Luxembourg Institute of Science and Technology, Esch-sur-Alzette, Luxembourg District, L-4362, Luxembourg; 3 Martel Innovate, Dübendorf, 8600, Switzerland; 4 Accelleran, Antwerpen, 2018, Belgium; 5 Walton Institute, Carriganore, X91 XD96, Ireland; 6 Politecnico di Bari, Bari, 70125, Italy; 7 Consiglio Nazionale delle Ricerche, Torino, 10129, Italy; 8 Universita degli Studi di Genova, Genova, 16129, Italy; 9 Consorzio Nazionale Interuniversitario per le Telecomunicazioni, Parma, Emilia-Romagna, 43124, Italy

**Keywords:** Sustainability, 6G Networks, Mobile Networks, Environmental Impact, Next-Generation Connectivity, Green Communications & ICT, Digital Transformation

## Abstract

As mobile networks evolve towards 6G, sustainability must be a central focus to address the environmental impacts of increasing energy consumption and resource use. This open letter highlights insights from the "Towards Sustainable 6G" workshop, where seven EU-funded projects - 6G4Society, BeGREEN, COALESCE, IN2CCAM, CENTRIC, 6Green, and 6G-TWIN - showcased innovative solutions for integrating energy efficiency, renewable energy, and ecological resilience into next-generation mobile networks. The projects emphasize AI-driven optimisation, sustainable infrastructure design, and cross-disciplinary collaboration as key strategies for reducing the ecological footprint of 6G systems. A joint statement underscores the necessity of embedding sustainability into 6G design, with follow-up activities planned to advance green telecommunications.

## Introduction

As mobile networks evolve from 5G to 6G and beyond, addressing the environmental impact of these systems becomes increasingly critical. The growing demand for data and connectivity means that energy consumption will continue to rise, and sustainability must become a central consideration in each new generation of network design. By embedding sustainable practices - such as energy efficiency, resource optimisation, and ecological resilience - into future networks, we can shape a digital infrastructure that supports both technological progress and environmental leadership.

**Figure 1.**
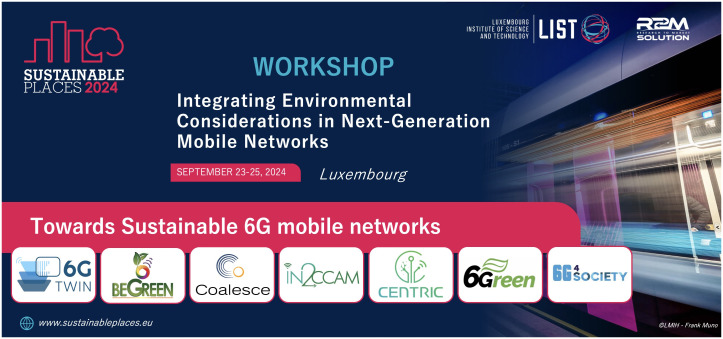
Sustainable Places 2024 Conference, Banner of the “Towards Sustainable 6G mobile networks” workshop held on 25 September 2024.

During the "Towards Sustainable 6G: integrating environmental considerations in next-generation mobile networks" workshop ( Figure 1), seven EU-funded projects came together to share innovative approaches and discuss green technologies and strategies aimed at reducing the ecological footprint of future mobile networks. These projects span a range of solutions from AI-driven network optimization to sustainable infrastructure design.

## Key contributions from participating projects

**6G4Society** is a Coordination and Support Action dedicated to ensuring that societal, environmental, and economic values are integrated into the design, development, and adoption of 6G technology. This multidisciplinary approach involves technologists, ethicists, legal experts, policymakers, and social scientists to ensure that 6G advances align with societal and environmental values.

*A Holistic Approach to Sustainability* - 6G4Society adopts a holistic approach to sustainability by addressing societal and environmental concerns through multi-stakeholder engagement. The project aims to create a unified framework for a value-based, sustainable, and ethical 6G ecosystem. This includes defining and promoting the use of Key Value Indicators (KVIs) and Key Sustainability Indicators (KSIs) to guide 6G development, in line with Europe's vision for a sustainable, inclusive, and human-centric digital future.

*Public Engagement for User-Centric 6G* - A key focus of 6G4Society is public engagement. By understanding end-users' needs, fears, and expectations, the consortium aims to develop a Technology Acceptance Model for 6G, fostering better understanding, acceptance and access to next generation networks. By collaborating with other SNS JU projects and key stakeholders in the telecommunications industry, such as the 6G-IA and NetWorld communities, 6G4Society envisions future 6G-based networks that not only meet technical requirements but also address social and environmental sustainability needs.

*Promoting Sustainability in 6G Networks* - 6G4Society is organizing workshops and webinars to explore sustainability challenges in mobile networks, particularly focusing on ecological sustainability, resource reduction, and reusability. By emphasizing the importance of integrating social considerations into 6G networks, the project aims to foster a sustainable future for 6G.

**BeGREEN** proposes a comprehensive solution for radio networks that goes beyond capacity expansion by integrating power consumption as a core metric. This project focuses on improving energy efficiency in the Radio Access Network (RAN), where most energy consumption in mobile networks occurs. By leveraging AI and machine learning innovations, BeGREEN seeks to optimize RAN performance while reducing energy use. One of the project’s key contributions is the integration of Open RAN technology, which offers more flexibility and allows for energy-efficient customization across different network environments.

The project emphasizes the importance of addressing energy consumption beyond just public 5G networks, expanding into use cases like private 5G networks for education, construction sites, hospitals, and manufacturing. These sectors require reliable, low-latency connectivity, but they also present opportunities to implement energy-saving measures. BeGREEN aims to balance this demand by developing energy-efficient solutions tailored to specific industries.

BeGREEN is contributing to ongoing global standards for energy efficiency in mobile networks. The project timeline includes significant milestones in network energy savings, aligning with international efforts to reduce the carbon footprint of ICT and mobile networks by 45% by 2030

**COALESCE** is at the forefront of driving innovation through the convergence of energy grids and telecommunication networks, leveraging a cross-optimization platform to seamlessly integrate energy efficiency across interconnected infrastructures. The project’s mission is to develop a holistic and groundbreaking framework that simultaneously optimizes data networks and microgrid infrastructures powered by renewable energy, aligning with the twin green and digital transitions central to Europe’s sustainability goals.

COALESCE addresses the critical challenge of managing energy consumption in the increasingly energy-intensive domains of computing, data processing, and communication—areas that are fundamental to the development of future 5G and 6G networks. The project’s vision is to transform these sectors by creating energy-efficient ICT networks and advanced computing architectures tailored for renewable-powered microgrids, while simultaneously enhancing the integration of these systems to foster resilient, smart, and sustainable communities.

The project’s core objectives include the development of pioneering energy-efficient network architectures, optimization of renewable energy provisioning through enhanced connectivity and data sharing across microgrids, and validation of these solutions through real-world use cases. These use cases span from in-building energy asset management and simultaneous wireless information and power transfer (SWIPT) in microgrids, to energy-efficient radio access networks for 5G/6G, and the integration of smart local energy systems with collaborative e-transportation networks. By leveraging cutting-edge technologies like SWIPT and advanced learning-based optimization models, COALESCE aims to significantly improve energy efficiency in wireless sensor networks and in-building energy management systems. Moreover, the project integrates state-of-the-art energy consumption models with next-generation RAN systems, ensuring efficient operation in future 5G and 6G environments, thus bridging the gap between telecommunications and energy sustainability.

**IN2CCAM** is advancing Connected, Cooperative, and Automated Mobility (CCAM) technologies by integrating these systems into fleet and traffic management, aiming to reduce congestion, improve safety, and decrease emissions. The project focuses on developing innovative services for connected and automated vehicles (CAVs) and infrastructures, enhancing mobility systems across Europe. IN2CCAM involves 21 partners from 10 countries, with demonstrations taking place in six living labs, including cities in Italy, France, Greece, and Spain.

The project tackles the transition phase where human-driven vehicles (HVs) and CAVs will coexist, developing traffic management strategies for mixed traffic environments. By implementing smart physical and digital infrastructures such as intelligent traffic lights, dynamic dedicated lanes, and integrated traffic management systems, IN2CCAM seeks to optimize traffic flow, reduce congestion, and improve the safety and efficiency of road transport. The project also addresses freight logistics, ensuring that automated and shared vehicle technologies can support efficient transport and reduce environmental impacts.

A key aspect of IN2CCAM’s approach is the integration of Artificial Intelligence (AI) to optimize traffic management in real-time. Technologies such as V2V (vehicle-to-vehicle) and V2I (vehicle-to-infrastructure) communication are used to manage intersections without traffic lights, enhancing the smoothness of traffic flow and reducing delays. IN2CCAM aims to achieve societal impacts, including fewer road accidents, lower transport emissions, and inclusive mobility solutions that benefit all citizens. By enhancing the interoperability of CCAM services and aligning them with public transport systems, the project ensures that future mobility services are sustainable and accessible to everyone.

**CENTRIC** is predicated upon applying AI techniques to the design of modular wireless connectivity frameworks that balance user and service requirements with environmental constraints, making sustainable network operation a priority. To this end, the project is developing an AI-based air interface (AI-AI) that adapts to specific user and application requirements, optimizing transceivers, waveforms, and resource management.

This approach ensures that network performance is tailored to real-time demands while maintaining a focus on sustainability factors like energy efficiency, CO2 emissions, and fairness in information distribution. CENTRIC emphasizes the integration of machine learning (ML) models within physical network nodes, ensuring that data processing is optimized based on available resources like CPUs and GPUs.

One of the unique aspects of CENTRIC is its human-centric approach, which focuses on ensuring that sustainability targets, such as minimizing electromagnetic field (EMF) exposure and reducing energy consumption, are integrated into the network’s core operations. This goes beyond treating sustainability as a constraint and positions it as a key objective of network management. CENTRIC’s AI-driven solutions are designed to operate within decision quality thresholds and deadlines, making them both scalable and efficient across diverse network environments.

**6Green** is developing a service-based, interconnected ecosystem that promotes energy efficiency across the entire 5/6G value chain. The project’s objective is to enable 5/6G networks and their vertical applications to significantly reduce their carbon footprint, targeting a reduction by a factor of 10 or more. To achieve this, 6Green exploits and extends state-of-the-art cloud-native technologies and service-based architectures (SBA), incorporating new cross-domain enablers that ensure flexibility, scalability, and sustainability for all stakeholders.

A key innovation of 6Green is the introduction of energy-aware backpressure mechanisms, which exploit information about the infrastructure-level energy and resource consumption through observability and analytics to assess the energy consumption and carbon footprint induced by vertical applications, network slices, and the overall 5/6G infrastructure. By processing and exposing this information at multiple levels, from edge-cloud infrastructure to individual applications, 6Green ensures that stakeholders — ranging from infrastructure providers to application developers — can make informed decisions to reduce energy use and receive incentives for taking these actions.

The project also aims to promote joint, sustainable behaviors through the development of Decarbonization Level Agreements (DLAs), which incentivize energy-efficient operations across the network. These agreements may be market-driven, where stakeholders self-regulate their carbon output, or enforced by regulatory bodies to ensure widespread adoption of sustainable practices. 6Green’s vision is to create a win-win scenario where economic success aligns with environmental responsibility, driving a green economy within the 5/6G ecosystem.

**6G-TWIN** aims to develop an AI-native 6G architecture that integrates Network Digital Twins (NDT) for enhanced network simulation, planning, operation, and management. The project addresses the pressing need for practical applications of network digital twin technology, bridging the gap between ambitious research concepts and the realities faced by the telecommunications industry.

By leveraging NDT, 6G-TWIN seeks to create tangible use cases that demonstrate the potential benefits of this innovative approach. One of the key applications of 6G-TWIN is related to connected and automated mobility, where NDT solutions are utilized to anticipate and predict network behavior for teleoperated vehicles before their journey begins. This predictive capability ensures that high-quality service and network resource availability are maintained throughout the vehicle’s path, enhancing safety and efficiency in connected mobility. Efficient energy management is of course crucial in this context, as it allows for sustainable operation by reducing power consumption across network resources and connected mobility systems.

Additionally, 6G-TWIN features an energy savings demonstrator that uses NDT solutions to optimize the network's energy efficiency in near real-time. By adapting network behavior dynamically, the project aims to achieve significant improvements in end-to-end energy consumption, reinforcing the commitment to sustainability within the 6G framework.

Through these demonstrators, 6G-TWIN not only showcases the practical implementation of NDT but also supports AI training and inference, facilitating comprehensive data generation and "what-if" analyses that will inform future network development. It is also essential to assess the efficiency and feasibility of deploying NDT to save energy, as the process itself, including data collection, processing, and AI operations, can introduce additional energy demands. Understanding this balance will be crucial to ensuring that the benefits of NDT justify its energy footprint and contribute positively to sustainable network practices.

## Joint statement on sustainability in 6G

The collaborative outcome of this workshop highlights that sustainability in 6G mobile networks is not an optional feature but a necessity. Future wireless communication systems must be designed with sustainability at their core, accounting for its different facets and leveraging cross-disciplinary methods and technologies. Such a challenge is made even more urgent by the dual role of AI and ML: on the one hand, they have the potential to greatly improve network efficiency; on the other, they represent themselves as a major contribution to energy consumption. By tackling the challenge from multiple perspectives—network optimisation, AI-driven automation, human-centric design, efficient AI/ML training and inference, and energy-communication integration—these projects demonstrate that a sustainable 6G is both possible and essential. This includes integrating renewable energy into network operations and optimizing both data networks and microgrids to ensure that sustainability is embedded into the infrastructure of future communication systems.

We, the authors, are committed to continuing this collaboration and advocating for sustainable solutions as part of the evolution of mobile networks. The insights gained from the workshop will be disseminated in further follow-up activities within the contributing projects.

## Conclusion

The future of mobile networks lies in the ability to innovate technologies and solutions while taking into account the main societal and environmental needs and challenges. The "Towards Sustainable 6G" workshop was an important step for several organisations involved both in the SNS JU initiative and in the Built4People partnership to align research and innovation efforts that are key to developing sustainable communication networks as the backbone of our digital society. In this respect, we encourage other stakeholders — researchers, policymakers, and industry leaders — to join us in embedding sustainability as a fundamental criterion for next-generation network design and development.

## Ethics and consent

Ethical approval and consent were not required.

## Disclaimer

The views expressed in this article are those of the author(s). Publication in Open Research Europe does not imply endorsement by the European Commission.

## Data Availability

No data are associated with this article.
