# Towards sustainable 6G: A collaborative call to action for addressing environmental challenges in (and thanks to) future mobile networks

**DOI:** 10.12688/openreseurope.18767.2

**Published:** 2025-09-19

**Authors:** Regis Decorme, Sébastien Faye, Monique Calisti, Simon Pryor, Indrakshi Dey, Maria Pia Fanti, Francesco Malandrino, Chiara Lombardo

**Affiliations:** 1 R2M Solution, Roquefort-les-Pins, 06330, France; 2 Luxembourg Institute of Science and Technology, Esch-sur-Alzette, Luxembourg District, L-4362, Luxembourg; 3 Martel Innovate, Dübendorf, 8600, Switzerland; 4 Accelleran, Antwerpen, 2018, Belgium; 5 Walton Institute, Carriganore, X91 XD96, Ireland; 6 Politecnico di Bari, Bari, 70125, Italy; 7 Consiglio Nazionale delle Ricerche, Torino, 10129, Italy; 8 Consorzio Nazionale Interuniversitario per le Telecomunicazioni, Parma, Emilia-Romagna, 43124, Italy; 9 Universita degli Studi di Genova, Genova, 16129, Italy

**Keywords:** Sustainability, 6G Networks, Mobile Networks, Environmental Impact, Next-Generation Connectivity, Green Communications & ICT, Digital Transformation

## Abstract

As mobile networks evolve towards 6G, sustainability must be a central focus to address the environmental impacts of increasing energy consumption and resource use. This open letter highlights insights from the "Towards Sustainable 6G" workshop, where seven EU-funded projects - 6G4Society, BeGREEN, COALESCE, IN2CCAM, CENTRIC, 6Green, and 6G-TWIN - showcased innovative solutions for integrating energy efficiency, renewable energy, and ecological resilience into next-generation mobile networks. The projects emphasize AI-driven optimisation, sustainable infrastructure design, and cross-disciplinary collaboration as key strategies for reducing the ecological footprint of 6G systems. A joint statement underscores the necessity of embedding sustainability into 6G design, with follow-up activities planned to advance green telecommunications.

## Introduction

As mobile networks evolve from 5G to 6G and beyond, addressing the environmental impact of these systems becomes increasingly critical. The growing demand for data and connectivity means that energy consumption will continue to rise, and sustainability must become a central consideration in each new generation of network design. By embedding sustainable practices - such as energy efficiency, resource optimisation, and ecological resilience - into future networks, we can shape a digital infrastructure that supports both technological progress and environmental leadership.

**Figure 1.**
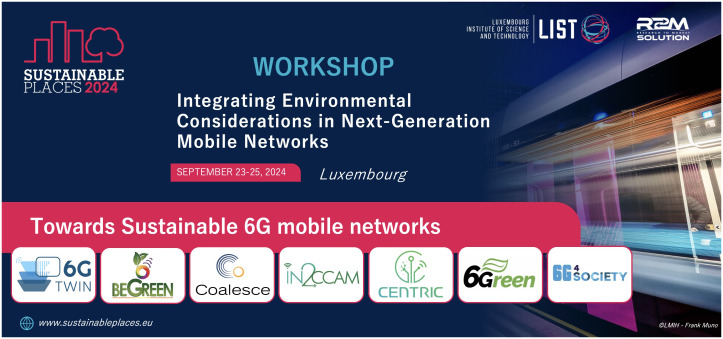
Sustainable Places 2024 Conference, Banner of the “Towards Sustainable 6G mobile networks” workshop held on 25 September 2024.

During the "Towards Sustainable 6G: integrating environmental considerations in next-generation mobile networks" workshop ( Figure 1), seven EU-funded projects came together to share innovative approaches and discuss green technologies and strategies aimed at reducing the ecological footprint of future mobile networks. These projects span a range of solutions from AI-driven network optimization to sustainable infrastructure design. This open letter does not aim to provide an exhaustive technical assessment of project results. Instead, it synthesises shared strategic approaches and early insights from projects at varying stages of implementation, highlighting how sustainability is being addressed from the outset in 6G research across Europe.

## Key contributions from participating projects

**6G4Society** is a Coordination and Support Action dedicated to ensuring that societal, environmental, and economic values are integrated into the design, development, and adoption of 6G technology. This multidisciplinary approach involves technologists, ethicists, legal experts, policymakers, and social scientists to ensure that 6G advances align with societal and environmental values.

*A Holistic Approach to Sustainability* - 6G4Society adopts a holistic approach to sustainability by addressing societal and environmental concerns through multi-stakeholder engagement. The project aims to create a unified framework for a value-based, sustainable, and ethical 6G ecosystem. This includes defining and promoting the use of Key Value Indicators (KVIs) and Key Sustainability Indicators (KSIs) to guide 6G development, in line with Europe's vision for a sustainable, inclusive, and human-centric digital future.

*Public Engagement for User-Centric 6G* - A key focus of 6G4Society is public engagement. By understanding end-users' needs, fears, and expectations, the consortium aims to develop a Technology Acceptance Model for 6G, fostering better understanding, acceptance and access to next generation networks. By collaborating with other SNS JU projects and key stakeholders in the telecommunications industry, such as the 6G-IA and NetWorld communities, 6G4Society envisions future 6G-based networks that not only meet technical requirements but also address social and environmental sustainability needs.

*Promoting Sustainability in 6G Networks* - 6G4Society is organizing workshops and webinars to explore sustainability challenges in mobile networks, particularly focusing on ecological sustainability, resource reduction, and reusability. By emphasizing the importance of integrating social considerations into 6G networks, the project aims to foster a sustainable future for 6G.

**BeGREEN** proposed a comprehensive solution for evolving radio networks that goes beyond capacity expansion by integrating power consumption as a core metric, focusing on energy efficiency in the Radio Access Network (RAN), where most energy in mobile networks is consumed. The project considered both economic and societal factors, linking energy cost and the necessary reduction in global emissions. By leveraging AI and machine learning innovations, BeGREEN optimized RAN performance while reducing energy use. A key contribution was the integration of Open RAN technology, providing a flexible, energy-efficient architecture across diverse network environments, including private 5G networks for education, hospitals, manufacturing, and construction sites.

Among BeGREEN’s major achievements, the BeGREEN Intelligence Plane ^1^ implemented an O-RAN-based architecture with a serverless execution environment hosting AI/ML models for rApps/xApps, enabling inference and training services directly in the RAN and Edge. The BeGREEN ISAC system, which can operate independently or in combination with Reconfigurable Intelligent Surfaces (RISs), extended RAN coverage in urban areas and enhanced sensing capabilities. Hardware acceleration innovations, including alternative ARM-based CU/DU processing architectures, reduced power consumption. Several of these solutions are being incorporated into commercial products, while others are being further explored in follow-on 6G R&I projects. The project also contributed to ongoing global standards for energy efficiency, aligning with international goals to reduce ICT and mobile network carbon footprints by 45% by 2030.

**COALESCE** focuses on the convergence of energy grids and telecommunication networks to address the environmental and operational challenges of future 6G systems. The core mission is to develop a holistic framework that simultaneously optimizes data networks and renewable energy-powered microgrid infrastructures, aligning with Europe's dual goals of green and digital transitions. This is achieved by leveraging a hierarchical computing architecture that includes edge, fog, and cloud layers. This architecture is designed to jointly minimize total latency and energy across all edge-generated tasks by intelligently offloading them to the most efficient computing tier. The methodology for this optimization involves a mathematical formulation and a greedy heuristic algorithm that considers system and network constraints and balances latency and energy costs ^2^. Preliminary simulations confirm the feasibility of this cross-optimization, with results showing a median energy saving of over 30% and improved bandwidth utilization compared to centralized or static allocation methods. The project's approach integrates cutting-edge technologies, such as Simultaneous Wireless Information and Power Transfer (SWIPT) and advanced learning-based optimization models, to enhance energy efficiency in wireless sensor networks and in-building energy management systems ^3^. This integration with next-generation Radio Access Network (RAN) systems is crucial for ensuring efficient operation in future 5G and 6G environments, thereby bridging the gap between telecommunications and energy sustainability ^4^.

The project’s core objectives include the development of pioneering energy-efficient network architectures, optimization of renewable energy provisioning through enhanced connectivity and data sharing across microgrids, and validation of these solutions through real-world use cases. These use cases span from in-building energy asset management and simultaneous wireless information and power transfer (SWIPT) in microgrids, to energy-efficient radio access networks for 5G/6G, and the integration of smart local energy systems with collaborative e-transportation networks. By leveraging cutting-edge technologies like SWIPT and advanced learning-based optimization models, COALESCE aims to significantly improve energy efficiency in wireless sensor networks and in-building energy management systems. Moreover, the project integrates state-of-the-art energy consumption models with next-generation RAN systems, ensuring efficient operation in future 5G and 6G environments, thus bridging the gap between telecommunications and energy sustainability.

**IN2CCAM** is advancing Connected, Cooperative, and Automated Mobility (CCAM) technologies by integrating these systems into fleet and traffic management, aiming to reduce congestion, improve safety, and decrease emissions. The project focuses on developing innovative services for connected and automated vehicles (CAVs) and infrastructures, enhancing mobility systems across Europe. IN2CCAM involves 21 partners from 10 countries, with demonstrations taking place in six living labs, including cities in Italy, France, Greece, and Spain.

The project tackles the transition phase where human-driven vehicles (HVs) and CAVs will coexist, developing traffic management strategies for mixed traffic environments. By implementing smart physical and digital infrastructures such as intelligent traffic lights, dynamic dedicated lanes, and integrated traffic management systems, IN2CCAM seeks to optimize traffic flow, reduce congestion, and improve the safety and efficiency of road transport. The project also addresses freight logistics, ensuring that automated and shared vehicle technologies can support efficient transport and reduce environmental impacts.

A key aspect of IN2CCAM’s approach is the integration of Artificial Intelligence (AI) to optimize traffic management in real-time. Technologies such as V2V (vehicle-to-vehicle) and V2I (vehicle-to-infrastructure) communication are used to manage intersections without traffic lights, enhancing the smoothness of traffic flow and reducing delays. IN2CCAM aims to achieve societal impacts, including fewer road accidents, lower transport emissions, and inclusive mobility solutions that benefit all citizens. By enhancing the interoperability of CCAM services and aligning them with public transport systems, the project ensures that future mobility services are sustainable and accessible to everyone.

**CENTRIC** is predicated upon applying AI techniques to the design of modular wireless connectivity frameworks that balance user and service requirements with environmental constraints, making sustainable network operation a priority. To this end, the project is developing an AI-based air interface (AI-AI) that adapts to specific user and application requirements, optimizing transceivers, waveforms, and resource management.

This approach ensures that network performance is tailored to real-time demands while maintaining a focus on sustainability factors like energy efficiency, CO2 emissions, and fairness in information distribution. CENTRIC emphasizes the integration of machine learning (ML) models within physical network nodes, ensuring that data processing is optimized based on available resources like CPUs and GPUs. Indeed, as also highlighted in the project’s deliverable D4.3 ^5^, combining AI with domain-specific knowledge and techniques offers much better performance, and results in lower resource consumption, than solely relying on AI.

One of the unique aspects of CENTRIC is its human-centric approach, which focuses on ensuring that sustainability targets, such as minimizing electromagnetic field (EMF) exposure and reducing energy consumption, are integrated into the network’s core operations. This goes beyond treating sustainability as a constraint and positions it as a key objective of network management. CENTRIC’s AI-driven solutions are designed to operate within decision quality thresholds and deadlines, making them both scalable and efficient across diverse network environments. Early results, published as deliverable D5.3 ^6^, include a list of key performance indicators and key value indicators (KPI, KVI) including both performance- and impact-related aspects of human-centric networking.

**6Green** is developing a service-based, interconnected ecosystem that promotes energy efficiency across the entire 5/6G value chain. The project’s objective is to enable 5/6G networks and their vertical applications to significantly reduce their carbon footprint, targeting a reduction by a factor of 10 or more. To achieve this, 6Green exploits and extends state-of-the-art cloud-native technologies and service-based architectures (SBA), incorporating new cross-domain enablers that ensure flexibility, scalability, and sustainability for all stakeholders.

A key innovation of 6Green is the introduction of energy-aware backpressure mechanisms, which exploit information about the infrastructure-level energy and resource consumption through observability and analytics to assess the energy consumption and carbon footprint induced by vertical applications, network slices, and the overall 5/6G infrastructure. By processing and exposing this information at multiple levels, from edge-cloud infrastructure to individual applications, 6Green ensures that stakeholders — ranging from infrastructure providers to application developers — can make informed decisions to reduce energy use and receive incentives for taking these actions.

In this respect, 6Green has produced an Observability Framework ^7^ able to ( *i*) retrieve energy consumption of hardware infrastructures; ( *ii*) break down the different contributions from any software instance/ microservice in the 5G infrastructure; as well as to ( *iii*) recombine these contributions to estimate the consumption levels ascribable to the virtual resources and artefacts consumed by upper level 5G stakeholders. The most noteworthy component of the framework is the Management Data Analytics Function (MDAF) ^8^, which has the ability to infer the power consumption ascribable to VMs/containers with a high-level accuracy.

The project also aims to promote joint, sustainable behaviors through the development of Decarbonization Level Agreements (DLAs), which incentivize energy-efficient operations across the network. These agreements may be market-driven, where stakeholders self-regulate their carbon output, or enforced by regulatory bodies to ensure widespread adoption of sustainable practices. 6Green’s vision is to create a win-win scenario where economic success aligns with environmental responsibility, driving a green economy within the 5/6G ecosystem.

Early results from 6Green include successful demonstrations of energy-aware backpressure mechanisms that dynamically reduce power consumption in virtualized edge 5G core networks.

**6G-TWIN** aims to develop an AI-native 6G architecture that integrates Network Digital Twins (NDT) for enhanced network simulation, planning, operation, and management. The project addresses the pressing need for practical applications of network digital twin technology, bridging the gap between ambitious research concepts and the realities faced by the telecommunications industry.

By leveraging NDT, 6G-TWIN seeks to create tangible use cases that demonstrate the potential benefits of this innovative approach. One of the key applications of 6G-TWIN is related to connected and automated mobility, where NDT solutions are utilized to anticipate and predict network behavior for teleoperated vehicles before their journey begins. This predictive capability ensures that high-quality service and network resource availability are maintained throughout the vehicle’s path, enhancing safety and efficiency in connected mobility. Efficient energy management is of course crucial in this context, as it allows for sustainable operation by reducing power consumption across network resources and connected mobility systems.

Additionally, 6G-TWIN features an energy savings demonstrator that uses NDT solutions to optimize the network's energy efficiency in near real-time. By adapting network behavior dynamically, the project aims to achieve significant improvements in end-to-end energy consumption, reinforcing the commitment to sustainability within the 6G framework.

Through these demonstrators, 6G-TWIN not only showcases the practical implementation of NDT but also supports AI training and inference, facilitating comprehensive data generation and "what-if" analyses that will inform future network development. It is also essential to assess the efficiency and feasibility of deploying NDT to save energy, as the process itself, including data collection, processing, and AI operations, can introduce additional energy demands. Understanding this balance will be crucial to ensuring that the benefits of NDT justify its energy footprint and contribute positively to sustainable network practices.

Early results at project mid-term include a multi-layered reference model and functional framework for NDT ^9^, a GDPR-compliant telemetry system, a graph-based digital twin, and advanced AI models for predictive and reactive control. Early integration efforts have prepared the two project demonstrators—teleoperated driving and energy efficiency, ensuring the architecture and tools are ready for implementation and testing during the upcoming phase.

## Positioning within broader sustainability frameworks

While energy and ecological sustainability were key topics of this workshop, several of the presented projects (notably 6G4Society and 6Green) are beginning to integrate social and economic sustainability dimensions. These include public engagement, inclusion, and ethical frameworks (6G4Society), as well as mechanisms like Decarbonisation Level Agreements (6Green) that align economic incentives with carbon footprint reduction. Such approaches resonate with triple bottom line (TBL) models, which advocate balancing environmental, social, and economic considerations in long-term innovation strategies. This is also aligned with key findings from the 6G SNS IA white paper “ *What societal values will 6G address ?*” ^10^, which identifies trust, inclusion, and digital sovereignty as central to socially sustainable 6G development. However, there remains a gap in exploring circular economy-driven business models, and more work is needed to systematically connect these efforts with established sustainability frameworks such as the UN SDGs ^11^, ITU-R M.2160 ^12^, BEREC sustainability indicators ^13^ , and Hexa-X II’s suggested drivers and goals for 6G ^14^. We recommend future initiatives and cross-project collaborations take a structured approach in aligning with these frameworks to ensure balanced, systemic sustainability impact.

## Joint statement on sustainability in 6G

The points raised in this joint statement reflect the specific angles and priorities explored by the contributing projects. This is not intended as a comprehensive sustainability framework for 6G, but rather a first-step alignment based on currently active work. While environmental dimensions (e.g. energy and emissions) are the most addressed, social and economic sustainability perspectives — such as inclusion, affordability, circular economy, and digital sovereignty — are acknowledged as areas requiring further coordination and integration.

Our shared view emerging from the workshop highlights the following sustainability priorities: *Environmental*: Energy efficiency, integration of renewables, network- and microgrid-level optimization. *Social*: Public trust, inclusive access, user engagement in design and adoption. *Economic*: Incentive mechanisms (e.g. DLAs), scalable business models for energy reduction, potential for circular economy innovation.

Future wireless communication systems must be designed with sustainability at their core, accounting for its different facets and leveraging cross-disciplinary methods and technologies. Such a challenge is made even more urgent by the dual role of AI and ML: on the one hand, they have the potential to greatly improve network efficiency; on the other, they represent themselves as a major contribution to energy consumption. By tackling the challenge from multiple perspectives—network optimisation, AI-driven automation, human-centric design, efficient AI/ML training and inference, and energy-communication integration—these projects demonstrate that a sustainable 6G is both possible and essential. This includes integrating renewable energy into network operations and optimizing both data networks and microgrids to ensure that sustainability is embedded into the infrastructure of future communication systems.

We, the authors, are committed to continuing this collaboration and advocating for sustainable solutions as part of the evolution of mobile networks. The insights gained from the workshop will be disseminated in further follow-up activities within the contributing projects.

## Challenges, convergence, and collaboration in sustainable 6G research

The integration of diverse technologies such as AI-driven optimization, renewable energy provisioning, digital twins, and cooperative mobility presents several challenges. These include managing heterogeneous infrastructures, ensuring real-time data coordination, aligning economic and environmental incentives, and overcoming organizational barriers to cross-project collaboration. Addressing these challenges requires robust coordination platforms, standardized interfaces, and flexible architectures that can adapt to evolving requirements. While each project targets specific aspects of sustainability and technology, their objectives are complementary and collectively support the overarching goal of sustainable 6G systems. Effective convergence depends on continuous dialogue, shared validation frameworks, and collaborative risk management. The multi-project consortium approach increases overall resilience by leveraging diverse expertise; however, the success of the collective effort depends on transparent communication, clear alignment of goals, and proactive mitigation of risks associated with delays or setbacks in individual projects.

## Recommendations and next steps

To accelerate progress towards sustainable 6G, we call on the research community, industry stakeholders, and policymakers to:

- Foster interdisciplinary collaborations bridging telecommunications, energy, AI, and social sciences.
- Develop shared metrics and standardised tools to evaluate sustainability across environmental, social, and economic dimensions.
- Support open platforms for joint experimentation, data sharing, and validation across projects.
- Encourage transparency and adaptability in project objectives to integrate new research groups and emerging technologies.

## Openness to new collaborations

The collaboration formed around the “Towards Sustainable 6G” workshop and its associated projects is open to expansion. We welcome interested research groups and stakeholders to engage with ongoing efforts. The evolving nature of 6G research ensures that project goals and strategies remain adaptable to incorporate new insights and partners, thereby strengthening the collective impact.

## Conclusion

The future of mobile networks lies in the ability to innovate technologies and solutions while taking into account the main societal and environmental needs and challenges. The "Towards Sustainable 6G" workshop was an important step for several organisations involved both in the SNS JU initiative and in the Built4People partnership to align research and innovation efforts that are key to developing sustainable communication networks as the backbone of our digital society.

In this respect, we encourage other stakeholders — researchers, policymakers, and industry leaders — to join us in embedding sustainability as a fundamental criterion for next-generation network design and development. This includes not only energy and ecological concerns, but also the social and economic pillars of sustainability. A more systematic application of models such as the UN SDGs ^11^ and TBL approaches, as well as structured monitoring via indicators such as those proposed by BEREC ^13^ and ITU-T L.1410 ^15^, will help ensure that 6G systems are sustainable in the broadest and most impactful sense.

## Ethics and consent

Ethical approval and consent were not required.

## Disclaimer

The views expressed in this article are those of the author(s). Publication in Open Research Europe does not imply endorsement by the European Commission.

## Data Availability

No data are associated with this article.
