# Supplementary material for: Towards sustainable 6G: A collaborative call to action for addressing environmental challenges in (and thanks to) future mobile networks
Source: Open Res Eur. 2025 Sep 19;4:260. Originally published 2024 Dec 9. [Version 2] doi: 10.12688/openreseurope.18767.2 (PMC12489451; doi:10.12688/openreseurope.18767.2)
Supplement: Peer review history

# Reviewer report: Reviewer response for version 2

**DOI:** 10.21956/openreseurope.23200.r60659

**Published:** 2025-10-01

**Authors:** Agbotiname Lucky Imoize

**Affiliations:** 1 University of Lagos, Akoka, Lagos, Nigeria

The authors have addressed my earlier comments satisfactorily. The letter has improved significantly and can be indexed in its current form.

Where applicable, are recommendations and next steps explained clearly for others to follow? (Please consider whether others in the research community would be able to implement guidelines or recommendations and/or constructively engage in the debate)

Partly

Does the article adequately reference differing views and opinions?

Yes

Are all factual statements correct, and are statements and arguments made adequately supported by citations?

No

Is the rationale for the Open Letter provided in sufficient detail? (Please consider whether existing challenges in the field are outlined clearly and whether the purpose of the letter is explained)

Yes

Is the Open Letter written in accessible language? (Please consider whether all subject-specific terms, concepts and abbreviations are explained)

Yes

Reviewer Expertise:

Wireless Communications, Wireless Security Systems, Artificial Intelligence.

I confirm that I have read this submission and believe that I have an appropriate level of expertise to confirm that it is of an acceptable scientific standard.

# Reviewer report: Reviewer response for version 1

**DOI:** 10.21956/openreseurope.20304.r48410

**Published:** 2025-03-04

**Authors:** Agbotiname Lucky Imoize

**Affiliations:** 1 University of Lagos, Akoka, Lagos, Nigeria

The letter provides valuable insights from the "Towards Sustainable 6G" workshop, where seven EU-funded projects—6G4Society, BeGREEN, COALESCE, IN2CCAM, CENTRIC, 6Green, and 6G-TWIN—showcased innovative solutions for integrating energy efficiency, renewable energy, and ecological resilience into next-generation mobile networks. The letter is concise and informative and highlights the key takeaway from the workshop. 

 1. Although the core objectives of the project, which include the development of pioneering energy-efficient network architectures, optimization of renewable energy provisioning through enhanced connectivity and data sharing across microgrids, and validation of these solutions through real-world use cases, are convincing, the real issues posed by the convergence of these emerging technologies, have not been given adequate coverage in the letter. The challenges ahead and probable solutions should be discussed briefly.

 2. It is not clear how the varying objectives of the individual projects will converge to meet the overall aim of the project as a whole. The likely hurdles to overcome to achieve this huge milestone should be elaborated briefly. 

 3. The interdependencies of the various projects should be critically analyzed. If one or more of the participating project partners fail, will the failure impact the overall objectives of the project?

 4. While I completely understand that this is a letter, a few references could be cited so the intended audience can learn more. 

 5. The action points/recommendations for next steps are not clearly explained for others to follow. We need to know whether others in the research community could successfully implement the reported guidelines or recommendations and/or constructively engage in the debate. 

 6. Is there room for more research groups to join the project, or is the door closed to newcomers? If more groups working on 6G-related research join the project, will any adjustments to the overall objective be made?

Where applicable, are recommendations and next steps explained clearly for others to follow? (Please consider whether others in the research community would be able to implement guidelines or recommendations and/or constructively engage in the debate)

Partly

Does the article adequately reference differing views and opinions?

Yes

Are all factual statements correct, and are statements and arguments made adequately supported by citations?

No

Is the rationale for the Open Letter provided in sufficient detail? (Please consider whether existing challenges in the field are outlined clearly and whether the purpose of the letter is explained)

Yes

Is the Open Letter written in accessible language? (Please consider whether all subject-specific terms, concepts and abbreviations are explained)

Yes

Reviewer Expertise:

Wireless Communications, Wireless Security Systems, Artificial Intelligence.

I confirm that I have read this submission and believe that I have an appropriate level of expertise to confirm that it is of an acceptable scientific standard, however I have significant reservations, as outlined above.

# Reviewer report: Reviewer response for version 1

**DOI:** 10.21956/openreseurope.20304.r48415

**Published:** 2025-01-22

**Authors:** Marja Matinmikko-Blue

**Affiliations:** 1 University of Oulu, Oulu, Finland

The document addresses a very timely and important topic. Thank you for putting it together!

 Key contributions section is an introduction of projects and their plans. It is not clear what the projects have actually done. Introductory texts can be found from the websites. Therefore, it is more interesting to read what the projects have actually done instead of what they plan to do.

 Joint statement on sustainability in 6G does not properly address environmental, social and economic sustainability perspectives and sustainable 6G and 6G for sustainability. It is a collection of some topics but it is not clear why those are important and why other critical topics are left out. To provide a joint statement on sustainability, it would be great to clearly state the statements considering the key sustainability dimensions and perspectives. 

 Overall, the topic is very important. More clarity on the projects' outputs would be helpful instead of introductions to projects. Additionally, joint statement on sustainability in 6G only addresses some sustainability aspects, which much be clearly stated that readers do not expect that this is a comprehensive sustainability in 6G statement.

Where applicable, are recommendations and next steps explained clearly for others to follow? (Please consider whether others in the research community would be able to implement guidelines or recommendations and/or constructively engage in the debate)

Partly

Does the article adequately reference differing views and opinions?

Partly

Are all factual statements correct, and are statements and arguments made adequately supported by citations?

Partly

Is the rationale for the Open Letter provided in sufficient detail? (Please consider whether existing challenges in the field are outlined clearly and whether the purpose of the letter is explained)

Yes

Is the Open Letter written in accessible language? (Please consider whether all subject-specific terms, concepts and abbreviations are explained)

Yes

Reviewer Expertise:

6G, sustainability, regulation, business, technology

I confirm that I have read this submission and believe that I have an appropriate level of expertise to confirm that it is of an acceptable scientific standard, however I have significant reservations, as outlined above.

# Reviewer report: Reviewer response for version 1

**DOI:** 10.21956/openreseurope.20304.r48789

**Published:** 2025-01-08

**Authors:** Seppo Yrjölä

**Affiliations:** 1 University of Oulu, Oulu, Finland

The manuscript summarized the key objectives and targeted contributions of seven EU-funded projects 6G4Society, BeGREEN, COALESCE, IN2CCAM, CENTRIC, 6Green, and 6G-TWIN. The paper discusses common strategies found in the  “Towards Sustainable 6G mobile networks” workshop 25 September 2024 for the sustainable 6G system development such as AI-driven optimization, sustainable infrastructure design, and cross-disciplinary collaboration.

 Discussion was very much focused on energy efficiency, renewable energy, and ecological resilience while the *social sustainability* goals and activities were only emerging. Similarly, third triple bottom line (TBL) corner the *sustainability in economics* considering balancing practices to create the long-term economic development with managing the social, environmental, and cultural aspects of the community was not visible? Sustainable business model innovation such as circular economy driven operational models with new business opportunities from combined footprint reduction and handprint improvements are essential parts of economic sustainability.

 Should be glad if you could discuss and position these research projects and their objectives and targeted contributions using the recent research literature and developed frameworks. Looking forward to reading the results of you assessment - Where is the focus, where do we have gaps - recommendations?

- UN SDGs, UN, “Global Sustainable Development Report: The Future is now - Science for Achieving Sustainable Development,” United Nations, 2019, https://sdgs.un.org/gsdr/gsdr2019.
- 6G SNS IA, “What societal values will 6G address?” May 2022, https://5g-ppp.eu/wp-content/uploads/2022/05/What-societal-values-will-6G-address-White-Paper-v1.0-final.pdf
- Hexa-X II, “Deliverable D1.1 Environmental, social, and economic drivers and goals for 6G,” June 2023, https://hexa-x-ii.eu/wp-content/uploads/2023/07/Hexa-X-II_D1.1_final-website.pdf.
- ITU-R, “Recommendation M.2160-0. Framework and overall objectives of the future development of IMT for 2030 and beyond,” Nov. 2023. https://www.itu.int/dms_pubrec/itu-r/rec/m/R-REC-M.2160-0-202311-I%21%21PDF-E.pdf
- Lüdeke-Freund F, et al., 2018 (Ref 1)
- BEREC, “Report on Sustainability Indicators for Electronic Communications Networks and Services, “Body of European Regulators for Electronic Communications (BEREC), BoR (23) 166, 2023, https://www.berec.europa.eu/en/document-categories/berec/reports/berec-report-on-sustainability-indicators-for-electronic-communications-networks-and-services.
- ITU-T L.1410 (11/2024), “Methodology for environmental life cycle assessments of information and communication technology goods, networks and services,” 2024 https://www.itu.int/ITU-T/recommendations/rec.aspx?rec=16010, https://www.itu.int/rec/dologin_pub.asp?lang=e&id=T-REC-L.1410-202411-P!!PDF-E&type=items

Where applicable, are recommendations and next steps explained clearly for others to follow? (Please consider whether others in the research community would be able to implement guidelines or recommendations and/or constructively engage in the debate)

Partly

Does the article adequately reference differing views and opinions?

Partly

Are all factual statements correct, and are statements and arguments made adequately supported by citations?

Partly

Is the rationale for the Open Letter provided in sufficient detail? (Please consider whether existing challenges in the field are outlined clearly and whether the purpose of the letter is explained)

Yes

Is the Open Letter written in accessible language? (Please consider whether all subject-specific terms, concepts and abbreviations are explained)

Yes

Reviewer Expertise:

Techno-economics, sustainability, future mobile communications, regulation and policy

I confirm that I have read this submission and believe that I have an appropriate level of expertise to confirm that it is of an acceptable scientific standard, however I have significant reservations, as outlined above.

## References

[1] : The sustainable business model pattern taxonomy—45 patterns to support sustainability-oriented business model innovation. *Sustainable Production and Consumption* .2018;15: 10.1016/j.spc.2018.06.004 145-162 10.1016/j.spc.2018.06.004
